# Crosstalk between the microbiome and the mucosal immunoglobulin A system in the lung, in health and disease

**DOI:** 10.3389/fcimb.2026.1796523

**Published:** 2026-04-10

**Authors:** Corentin Stavart, Sophie Gohy, Sarah Lebeer, Françoise Van Bambeke, Aurélie Crabbé, Charles Pilette

**Affiliations:** 1 Pole of Lung, Nose and Skin research (LUNS), Institute of Experimental and Clinical Research (IREC), Université catholique de Louvain (UCLouvain), Brussels, Belgium; 2 Department of Pediatrics, Cliniques universitaires Saint-Luc, Brussels, Belgium; 3 Department of Pulmonology, Cliniques universitaires Saint-Luc, Brussels, Belgium; 4 Cystic Fibrosis Reference Centre, Cliniques universitaires Saint-Luc, Brussels, Belgium; 5 Department of Bioscience Engineering, University of Antwerp, Antwerpen, Belgium; 6 Cellular and Molecular Pharmacology Laboratory, Louvain Drug Research Institute (LDRI), Université catholique de Louvain, Brussels, Belgium; 7 Laboratory of Pharmaceutical Microbiology (LPM), Ghent University, Ghent, Belgium

**Keywords:** airway epithelium, barrier dysfunction, chronic lung diseases, host–microbial interactions, lung microbiota, mucosal immunity, secretory IgA

## Abstract

The lung, and more specifically the airway epithelium, is continuously exposed to a wide range of inhaled environmental agents. Acting as a frontline physical and biochemical barrier, the airway epithelium orchestrates early host defense mechanisms, among which immunoglobulin A (IgA) plays a central role. Long considered sterile, the healthy lung is now recognized as a complex mucosal ecosystem harboring diverse and dynamic microbial communities, including bacteria, fungi, viruses, and archaea. Although the lung microbiome is generally transient and low in biomass, accumulating evidence suggests that it contributes to pulmonary homeostasis by supporting immune system maturation, preserving structural tissue integrity, and limiting pathogen colonization. How immune homeostasis is maintained in this constantly challenged environment remains however a central and largely unanswered question. This review synthesizes current state-of-the-art knowledge on the origin, composition, and functional determinants of the lung microbiome, with a specific focus on its bidirectional interplay with secretory IgA. We discuss microbiota-specific IgA responses, factors influencing IgA–microbiome interactions, and how these processes are disrupted in chronic and inflammatory lung diseases. Finally, we highlight major knowledge gaps and explore emerging therapeutic perspectives targeting IgA–microbiome crosstalk to restore pulmonary immune homeostasis.

## 1 Introduction

The lung, and more particularly the airway epithelium, is constantly exposed to a wide range of external agents such as pathogens, toxins, pollutants, and allergens. The airway epithelium acts as a frontline physical and biochemical barrier that relies on a multifaceted system, including mucociliary clearance and antimicrobial molecules such as lysozyme, antiproteases, defensins, lactoferrin, collectins, as well as immunoglobulin (Ig) A.

Beyond the role of this first line of defense against pathogens, the same protective mechanisms may also shape the composition of the lung microbiome. While healthy lungs were long considered sterile, their mucosal site is now recognized as a ecosystem containing very diverse microbial communities, including bacteria, fungi, viruses and archaea. Although the lung microbiome is generally viewed as transient and dynamic, shaped by clearance mechanisms such as coughing and mucociliary clearance, it likely plays a role in pulmonary homeostasis, contributing to immune system development, maintaining structural tissues integrity, and preventing pathogen colonization notably through competitive exclusion. Established early in life, its composition evolves over time and is influenced by various host and environmental factors, highlighting its intricate relationship with lung immunity and homeostasis.

In recent years, host–microbiome interactions have emerged as key regulators of mucosal immunity in the lung. How immune homeostasis is maintained in this constantly challenged environment remains however a central and largely unanswered question. Recent evidence highlights IgA as a pivotal mediator at this interface, both shaping microbial communities and being shaped by them. This review explores the current state-of-the-art knowledge on bidirectional regulatory circuits between mucosal host IgA immunity and microbiome in the lung and their alterations in disease, as well as current scientific gaps and putative therapeutic perspectives.

## 2 The old dogma “a healthy lung is sterile”

Historically, the lung was regarded as sterile organ. This paradigm was rooted in century-old research, with Lister’s pioneering experiments in 1868 (Lister, 1868). His comparison of open and closed rib fractures, where only the open fractures became infected, led him to propose that the upper respiratory tract must filter germs, preventing them from reaching the lungs. In 1888, Straus demonstrated that inhaled air containing bacteria did not lead to bacterial contamination of exhaled air, supporting the role of the upper respiratory tract as a filter, trapping solid particles (Straus, 1888). This was further suggested the same year by Hildebrand’s study on excised nasal and tracheal mucosa of rabbits, showing that air appeared entirely cleared of germs before reaching the trachea (Hildebrant, 1888), a finding also supported by Thomson in 1895 (Thomson and Hewlett, 1895).

Despite the prevailing paradigm of pulmonary sterility, early evidence of a respiratory microbiota can already be traced back to Béco’s work in 1899, which identified “conventional respiratory pathogens without invasion” in 23 lung autopsies (Béco, 1899). His findings were based on earlier doubts raised by Polguère, Claisse and Dürck, who conducted bacteriological analyses of the bronchi and lungs of children using aerobic culture-based methods available at that time (Polguère, 1888; Claisse, 1893; Dürck, 1897). Their research revealed a sparse “mixed bacterial flora” in the lung, also referred to as “latent microbism”.

Nevertheless, several factors contributed to the persistence of the sterility paradigm. These include: (a) culture-based methods primarily targeting known respiratory (aerobic) pathogens, overlooking potential resident microbial communities and anaerobic pathogens; (b) the presence of bacteria in samples from healthy individuals was often (mis)interpreted as contamination from the oropharyngeal flora; (c) bronchopulmonary sampling was mainly performed in the context of acute or chronic infections, reinforcing the perception that bacteria were only present during disease states; (d) the role of mucus, mucociliary clearance and alveolar macrophages in bacterial elimination further strengthened the idea of the lungs as “clean and sterile”.

Although debates on this concept persisted throughout the 20th century, a definitive paradigm shift occurred in 2010, with the advent of high-throughput sequencing technologies pioneered by Hilty’s metagenomic study (Hilty et al., 2010). As a result, the traditional binary view of sterility in health versus pathogenic colonization in disease evolved into a more nuanced understanding, recognizing the lung microbiome as an integral component of lung physiology in both health and disease.

## 3 The microbiome of healthy lungs

Since the early 2000s, the study of the microbiota, the community of microorganisms inhabiting a specific site of the human body (such as the gut, skin, or oral cavity), has become central in microbial ecology and human health research. The microbiome encompasses these microorganisms to include their collective genomes and functions as well as the environmental context. This holistic view highlights the microbiome as a dynamic ecosystem whose genetic and metabolic activities significantly influence host tissue physiology, immune responses, and even social, affective or cognitive behaviors. Understanding this intricated host-microbe relationship has shifted our perspective: human health is not solely a product of our own genome but could also be deeply intertwined with that of our microbial partners.

### 3.1 Origin and impact in the early years of life

Human lung development is a complex, multi-stage process that begins around day 21–24 of gestation with the formation of two respiratory buds from the ventral wall of the primitive foregut (Cardoso and Lü, 2006). These buds rapidly divide into the main bronchi, airway branching being mostly complete by 16 weeks (Gould, 1993). As development progresses, lung structures expand, air-blood barrier forms, epithelial cells differentiate, and surfactant begins to be produced. Alveolar formation starts in a late fetal stage and continues during early childhood up to approximately 3 years of age (Schittny, 2017), alongside other key maturational changes in the respiratory system (airway growth and modeling, chest wall and respiratory muscles development, maturation of respiratory control). Importantly, lung maturation and refinement persist beyond childhood, extending into young adulthood (Schittny, 2017), with lung function reaching its peak around age 20-25.

In parallel with structural maturation, emerging evidence, demonstrated through complementary approaches including culture-based assays, nucleic acid sequencing, histology, immunofluorescence, and microscopy, that microbial colonization of the respiratory tract may begin *in utero* (Al Alam et al., 2020; Mishra et al., 2021). Although the existence of a true placental microbiome remains controversial (Blaser et al., 2021), with several studies reporting no detectable (or indistinguishable from contaminants) microbial communities in the placenta (reviewed in (Kennedy et al., 2023; Panzer et al., 2023; Banchi et al., 2024)), and others demonstrating the presence (or trace, due to low biomass) of microbial (bacterial, viral, fungal and archaeal) DNA in amniotic fluid (Bearfield et al., 2002; Baschat et al., 2003; Rautava et al., 2012; Collado et al., 2016; Urushiyama et al., 2017; Wang et al., 2018; Zhu et al., 2018; Liu et al., 2019; Stinson et al., 2019a; He et al., 2020; Turunen et al., 2021; Campisciano et al., 2023; Kaisanlahti et al., 2023; Xu et al., 2025), fetal meconium (Baschat et al., 2003; Schultz et al., 2004; Biasucci et al., 2008; Jiménez et al., 2008; Dominguez-Bello et al., 2010; Mshvildadze et al., 2010; Koenig et al., 2011; Arboleya et al., 2012; Madan et al., 2012b; Rautava et al., 2012; Gosalbes et al., 2013; Hu et al., 2013; Makino et al., 2013; Moles et al., 2013; Ardissone et al., 2014; Del Chierico et al., 2015; Dong et al., 2015; Hansen et al., 2015; Chernikova et al., 2016; Chu et al., 2016; Collado et al., 2016; Nagpal et al., 2016; Wampach et al., 2017; Ferretti et al., 2018; Shi et al., 2018; Wang et al., 2018; Liu et al., 2019; Stinson et al., 2019a; Stinson et al., 2019b; Younge et al., 2019; He et al., 2020; Rackaityte et al., 2020; Turunen et al., 2021; Kang et al., 2022; Liu et al., 2022), placenta (Bearfield et al., 2002; Steel et al., 2005; Onderdonk et al., 2008; Jones et al., 2009; Satokari et al., 2009; Rautava et al., 2012; Stout et al., 2013; Aagaard et al., 2014; Cao and Mysorekar, 2014; Doyle et al., 2014; Antony et al., 2015; Dong et al., 2015; Zheng et al., 2015; Bassols et al., 2016; Collado et al., 2016; Gomez-Arango et al., 2017; Parnell et al., 2017; Zhu et al., 2018; Liu et al., 2019; Seferovic et al., 2019; Tuominen et al., 2019; Younge et al., 2019; Al Alam et al., 2020; Mishra et al., 2021; Turunen et al., 2021; Campisciano et al., 2023; Yang et al., 2024), as well as in fetal lung tissue as early as 11 weeks of gestational age (Al Alam et al., 2020; Mishra et al., 2021).

Although studying *in utero* microbiota is technically challenging, notably given the high risk of contamination from high−biomass sites such as the vagina, some studies have argued that putative *in utero* microbiota show remarkable consistency across body sites, although slight geographical and niche-specific variations have been observed. Despite their low abundance, these micro-organisms may exhibit metabolic activity, show gestational age-related variation, and appear unaffected by mode of delivery. Moreover, viable bacteria have also been isolated from some of these samples (Bearfield et al., 2002; Schultz et al., 2004; Steel et al., 2005; Jiménez et al., 2008; Makino et al., 2013; Moles et al., 2013; Collado et al., 2016; Wang et al., 2018; Zhu et al., 2018; Stinson et al., 2019b; Tuominen et al., 2019; Mishra et al., 2021; Turunen et al., 2021), with some cultivable only under conditions mimicking the fetal environment, further supporting the possibility of early microbial exposure before birth.

However, definitive evidence for a viable, active, and functioning microbiota *in utero* during healthy pregnancies is still lacking. Current evidence more convincingly supports that maternal microbiome-derived metabolites – rather than live microbes - cross the placental barrier and reach the fetus (Li et al., 2020; Wang et al., 2025). Since fetal lungs are filled with amniotic fluid during gestation, this suggests that antenatal lung development occurs in the presence of these microbial signals. At birth, microbial colonization accelerates rapidly, with bacteria detected in the nasopharynx and oral cavity within minutes (Wang et al., 2018) or in tracheal aspirates (Dominguez-Bello et al., 2010). Remarkably, by the age of two months, the lung bacterial microbiota already reaches an adult-like taxonomic composition (Biesbroek et al., 2014; Pattaroni et al., 2018; Gallacher et al., 2020).

### 3.2 Composition

The healthy human lower respiratory microbiota is an extremely low-biomass ecosystem (∼10^2–^10^4^ organisms/ml of lower-airway secretions), in stark contrast to the gut (∼10^11^ - 10^13^/g of feces) or oral cavity (∼10^9^/ml of saliva). This low microbial burden poses important methodological challenges, influences sampling strategies and notably increases the risk of contamination inherent to low-biomass sequencing, a critical issue in lung microbiome studies. Multiple approaches are used to characterize the lower airway microbiome, including sputum, tracheal aspiration, protected specimen brush and bronchoalveolar lavage (BAL), thereby challenging cross-study comparisons. Sputum sampling is non-invasive, easily repeatable and minimally diluted, but more prone to oropharyngeal contamination, whereas BAL is invasive and inherently diluted; nevertheless, no consistent differences in microbial diversity have been reported between both (Karim et al., 2025). In contrast to other organ microbiomes, the respiratory tract microbiota is relatively homogenous across lobes, making segmental sampling generally unnecessary (Charlson et al., 2011; Charlson et al., 2012a; Dickson et al., 2015).

While the microbial load may increase in acute infections such as pneumonia (∼10^7^/mL) and chronic respiratory diseases such as cystic fibrosis (CF), non-CF bronchiectasis (∼10^7^/mL), chronic obstructive pulmonary disease (COPD) or idiopathic pulmonary fibrosis (IPF) (10^5^/mL) (Macpherson and Harris, 2004; Sender et al., 2016; Dickson et al., 2017; Schneeberger et al., 2019; Pérez-Cobas et al., 2023a), the lung microbiota exists in a dynamic state shaped by a balance between microbial immigration and elimination. Microorganisms reach the lungs primarily through subclinical, repeated microaspirations of oropharyngeal and gastric contents, a process that occurs in healthy individuals, and exacerbated in patients with gastroesophageal reflux (Gleeson et al., 1997; Krishnan et al., 2020). In addition, direct spread along anatomically contiguous mucosal surfaces, particularly from the oral cavity and upper airways, substantially contributes to microbial seeding (Bassis et al., 2015; Venkataraman et al., 2015). In most cohorts studied to date, these niches appear to be largely dominated by *Streptococcus*, *Haemophilus* and *Prevotella* in the oral cavity; *Corynebacterium*, *Streptococcus*, *Cutibacterium* and *Dolosigranulum* in the nasopharynx; and *Streptococcus*, *Veillonella*, and *Prevotella* in the oropharynx (Bassis et al., 2015; Odendaal et al., 2024; Quinn-Bohmann et al., 2024), although these patterns vary with geography, lifestyle, host genetics and environmental exposures. These niches also harbor resident fungi such as *Candida*, as well as a rich repertoire of viruses (bacteriophages, *Herpesviridae, Papillomaviridae, Anelloviridae* and *Redondoviridae*) and archaea (*Methanosphaera*, *Methanobrevibacter*) (Ghannoum et al., 2010; Pride et al., 2012; Wylie et al., 2014; Abbas et al., 2019; Pausan et al., 2019). Consistent with the transient nature of the lung microbiome described above, these microbial inputs are counterbalanced by intrinsic protective mechanisms of the airways such as mucociliary clearance, cough, and innate and adaptative host defenses. In addition, local unique factors including oxygen tension, mucosal pH, nutrient availability, temperature, high surfactant levels and immune surveillance further determine which microbes may persist for longer periods (Dickson et al., 2014a). This tightly regulated equilibrium maintains a low microbial density, likely to preserve the lungs’ primary function of efficient gas exchange.

To better understand the variability observed between (healthy) individuals and over time, the respiratory microbiome is often conceptualized as comprising two distinct components, namely the “core” and “satellite” microbiomes (Hanski, 1982; Neu et al., 2021). The core microbiome refers to the set of dominant microbial species that are shared among healthy individuals. Thus, although the lung microbiome is inherently transient due to continuous microbial immigration and clearance, a reproducible core community can still be identified at the population level. In contrast, the satellite microbiome consists of less prevalent microbial species that exhibit rapid, reversible fluctuations in response to various pathophysiological and environmental factors.

**Figure 1:**
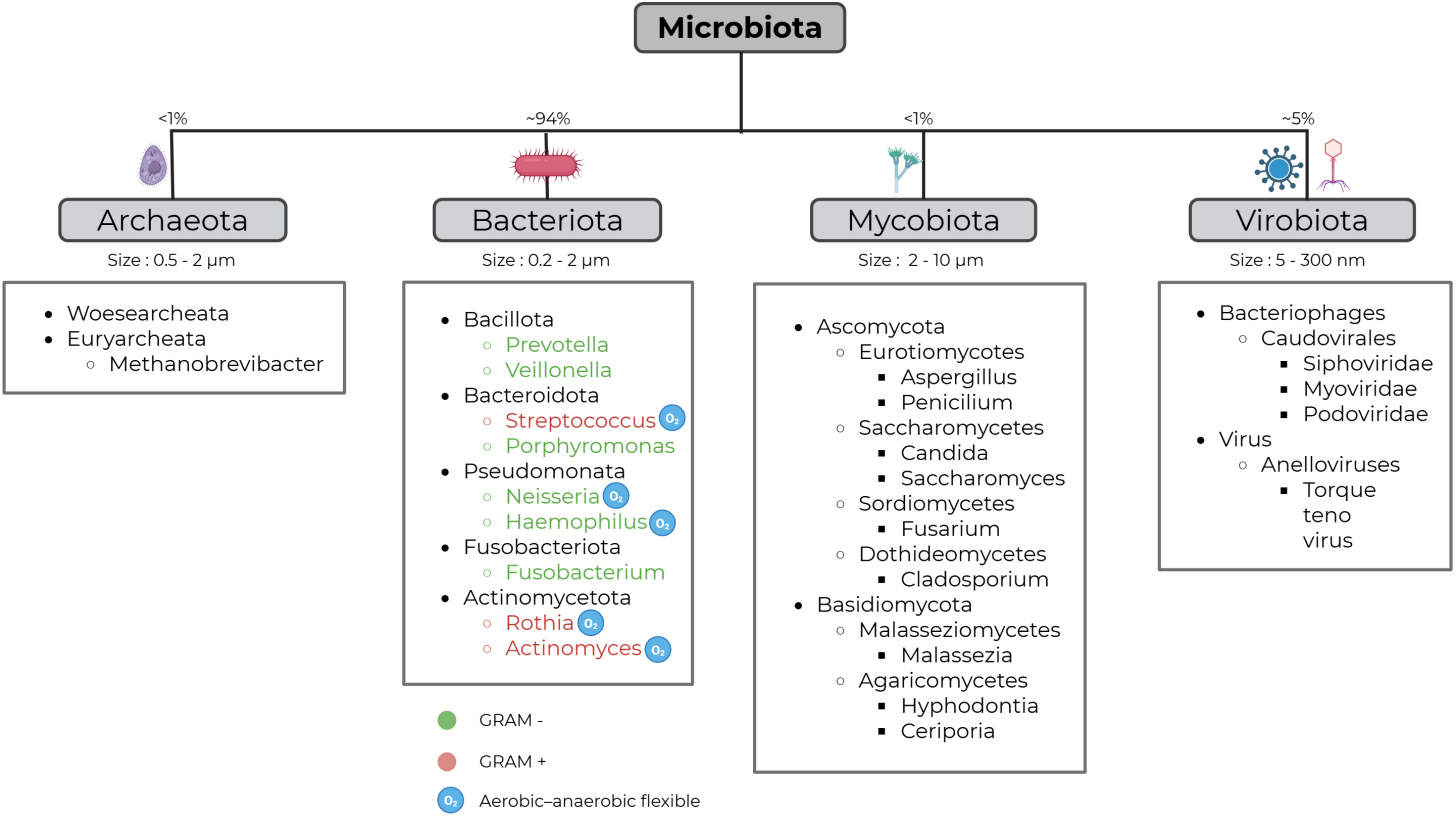
The healthy human core respiratory microbiota. The healthy lung harbors a dynamic, low-biomass microbiota of bacteria, fungi, viruses, and archaea. The relative proportions shown represent the distribution of total microbial DNA, and do not reflect microbial viability or metabolic activity. Continuous immigration (microaspiration, contiguous mucosal spread) and clearance (coughing, ciliary movement, alveolar macrophages, surfactant) shape communities, resulting in a transient yet reproducible core microbiome as demonstrated by studies using sputum, bronchoalveolar lavage, and protected brush samples (Willner et al., 2009; Hilty et al., 2010; Charlson et al., 2011; Charlson et al., 2012a; Charlson et al., 2012b; van Woerden et al., 2013; Cui et al., 2015; Dickson et al., 2015; Young et al., 2015; Lim et al., 2016; Abbas et al., 2017; Dickson et al., 2017; Koskinen et al., 2017; Mac Aogáin et al., 2018; Sharma et al., 2019; Haldar et al., 2020; Hassani et al., 2020; Huang et al., 2020; Rick et al., 2020; Soret et al., 2020; van Tilburg Bernardes et al., 2020; Ali et al., 2021; Choi et al., 2021; Martinsen et al., 2021; Tiew et al., 2021; Sachdeva and Das, 2022; Conradie et al., 2024; Semmler et al., 2024; Zhang et al., 2025). Created with Biorender.

The healthy core respiratory bacteriome (**Figure 1**) is predominantly composed of key genera such as *Prevotella*, *Veillonella*, and *Streptococcus*, with smaller proportions of *Porphyromonas*. Other frequently encountered genera include *Neisseria* and *Haemophilus*, whereas *Fusobacterium*, *Rothia* and *Actinomyces* are found in lower abundance (Hilty et al., 2010; Charlson et al., 2011; Dickson et al., 2015; Lim et al., 2016; Dickson et al., 2017; Haldar et al., 2020). Most members of the respiratory bacteriome are aerobic or facultatively anaerobic, although some genera, such as *Veillonella, Prevotella, Porphyromonas*, and *Fusobacterium*, are strict anaerobes.

In contrast to bacteria, the other components of the respiratory microbiome remain far less well characterized. Nevertheless, complex interkingdom crosstalk occurs among all these different microbial communities (see point 3.3 below). The healthy lung virome is dominated by bacteriophages, mainly from the Caudovirales order (Siphoviridae, Myoviridae, Podoviridae). These bacteriophages, or phages, are ubiquitous obligate viruses that infect and replicate within bacteria, whilst some specific viruses also parasitize archaea and fungi (Kinsella et al., 2022; Li et al., 2022). Because of this tight ecological relationship, viral composition is closely aligned with the microbiome present in each specific anatomical niche. Eukaryotic viruses are present in smaller numbers, primarily anelloviruses such as *Torque teno virus*, while herpesviruses are occasionally detected (Willner et al., 2009; Young et al., 2015; Abbas et al., 2017; Choi et al., 2021; Conradie et al., 2024). This virome is typically of low abundance, and its expansion in immunocompromised or chronically diseased individuals highlights the role of immune competence in maintaining viral equilibrium, a feature increasingly investigated as a plasma biomarker of immune status in transplant medicine (Jaksch et al., 2018).

The mycobiome, the fungal component of the microbiome, is thought to represent approximately 0.1% of the total microbiome (Qin et al., 2010). Despite this low abundance, the larger cell size of fungi and their involvement in a wide range of metabolic processes may suggest functional relevance (Underhill and Iliev, 2014; Iliev and Cadwell, 2021). The healthy lung mycobiome is mainly composed of the phyla *Ascomycota* and *Basidiomycota*, with high inter-individual variability. Among Ascomycota, key classes include Eurotiomycetes (*Aspergillus*, *Penicillium*) Saccharomycetes (*Candida*, *Saccharomyces*), Sordiomycetes (*Fusarium*) and Dothideomycetes (Cladosporium). Within Basidiomycota, Malasseziomycetes (*Malassezia*) and Agaricomycetes (*Hyphodontia, Ceriporia*) are notable (Charlson et al., 2012a; Charlson et al., 2012b; van Woerden et al., 2013; Cui et al., 2015; Mac Aogáin et al., 2018; Sharma et al., 2019; Huang et al., 2020; Rick et al., 2020; Soret et al., 2020; van Tilburg Bernardes et al., 2020; Ali et al., 2021; Martinsen et al., 2021; Tiew et al., 2021; Sachdeva and Das, 2022; Semmler et al., 2024; Zhang et al., 2025).

Finally, archaea are single-cell microorganisms with distinct cellular structures and metabolic pathways. They are present in very low numbers in human lungs, and their role is largely unexplored. Limited data suggest Woesearchaeata may be predominant in healthy lungs (Koskinen et al., 2017), alongside a few methanogenic archaea, such as *Methanobrevibacter* (Hassani et al., 2020).

### 3.3 Functions

**Figure 2:**
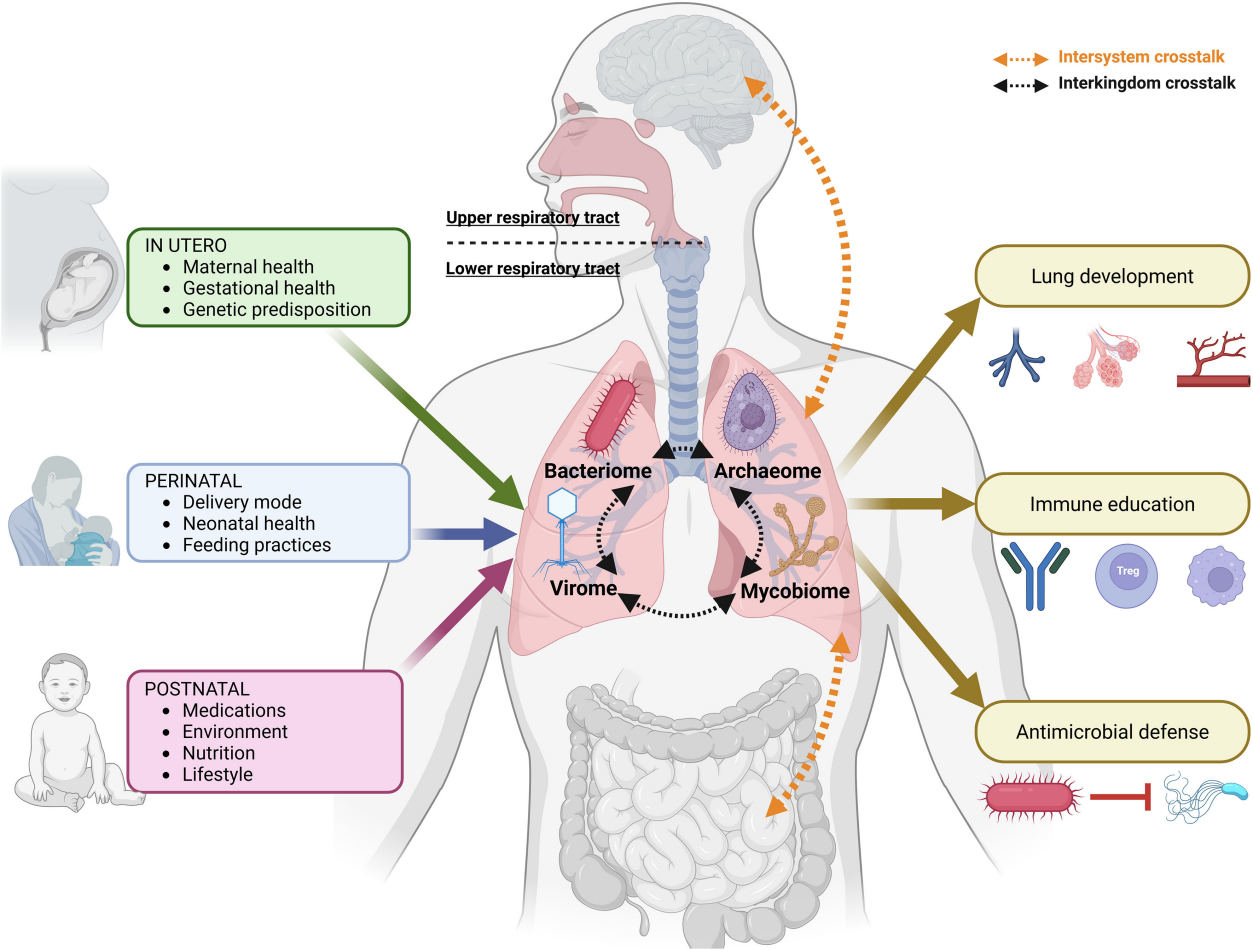
Schematic view of major determinants and functions of the respiratory microbiome in healthy human lungs. Left, key factors shaping the respiratory microbiome across development (in utero, perinatal, and postnatal). Right (brown), main functions of the respiratory microbiome. Based on references (Ben-Hur et al., 2004; Peterson et al., 2007; Bansal et al., 2010; Hapfelmeier et al., 2010; Herbst et al., 2011; Yun et al., 2014; Koppen et al., 2015; Schuijs et al., 2015; Edwards et al., 2020; Marquant et al., 2021; Schlosser-Brandenburg et al., 2021; Wu et al., 2021; Yang et al., 2022; Abushawish et al., 2023; Barfod et al., 2023; Pérez-Cobas et al., 2023b; Ge et al., 2025; Peneva et al., 2025; Pirr et al., 2025). Created with Biorender.

The respiratory microbiome plays three major roles, namely supporting lung structural development, educating the immune system, and providing defense against pathogens (**Figure 2**). The maternal microbiota has a major (direct or indirect) impact on fetal gene expression and DNA methylation, notably on genes essential for the fetal immune and nervous systems, protein synthesis and energy metabolism, as well as on epithelial barrier maturation, host-microbe interactions, and nutrient absorption. These effects are organ-specific, gestational age-dependent, and sex-dependent, with stronger impact in males (Gomez de Agüero et al., 2016; Li et al., 2020; Pessa-Morikawa et al., 2022; Husso et al., 2023; Gustafson et al., 2024; Wang et al., 2025). While this evidence comes across various fetal organs, specific insights into the developing lungs remain limited.

Throughout life, several factors may influence the respiratory microbiome (**Figure 2**). They can be broadly categorized into those acting *in utero*, at birth and during postnatal development or in later life. These include maternal health factors such as nutrition, tobacco and antibiotic use, as well as gestational conditions (e.g. placental disorders, gestational diabetes); early neonatal factors including delivery mode, neonatal health, and feeding practices; and postnatal influences such as environmental and lifestyle exposures, medication use, and chronic conditions throughout childhood. Their long-term impact, as well as the extent to which each factor contributes to shaping the respiratory microbiome, remains elusive at this stage (Koppen et al., 2015; Abushawish et al., 2023; Ge et al., 2025; Peneva et al., 2025; Pirr et al., 2025).

#### 3.3.1 Pulmonary development

Although numerous factors may shape the respiratory microbiome across the lifespan, microbial impacts on lung development begin before birth, mediated by the respiratory tract itself and by maternal gut–derived metabolites that reach the fetus *in utero*. *In vivo* gene expression differences are linked to microbially modulated metabolites, which are primarily derived from microbial or host-microbial amino acid and energy metabolism pathways already biologically and functionally active *in utero* (Aagaard et al., 2014; Antony et al., 2015; Wang et al., 2018; Liu et al., 2022). These include aromatic hydrocarbons, fatty acid derivatives (short-chain fatty acids (SCFA)), amino acid derivatives (tryptophan, dipeptides, 5-aminovaleric acid betaine, spermidine, putrescine, trimethylamine N-oxide), bile acid (coprocholic acid) and nucleobase derivatives, and probably many others to be identified (Liu et al., 2022; Pessa-Morikawa et al., 2022; Wang et al., 2025; Xu et al., 2025).

Lung organogenesis results from tightly regulated molecular and cellular interactions throughout gestation. These precise temporal and spatial processes rely on the dynamic activity of key developmental pathways, including Fibroblast Growth Factor (FGF), Retinoic Acid (RA), Sonic Hedgehog (SHH), Wingless-related Integration Site (WNT), Transforming Growth Factor β (TGF-β), Bone Morphogenetic Protein (BMP), Notch and Hippo signaling (Schittny, 2017; Caldeira et al., 2021). Their functions extend far beyond lung morphogenesis, regulating a wide range of homeostatic and pathogenic processes in adult tissues.

Interestingly, it has been shown that several microbial metabolites can regulate these signaling networks, both locally within the respiratory tract and systemically via metabolites produced by the maternal gut microbiota during pregnancy or the infant gut microbiota after birth. These pathways may therefore impact lung development, as detailed below. However, it is important to note that evidence for systemic signaling remains limited and is currently supported primarily by experimental studies in animal models. Germ-free mice and piglets, for instance, exhibit impaired parenchymal and alveolar formation, resulting in fewer but larger alveoli, reduced capillary angiogenesis, and altered surfactant gene expression and function (Yun et al., 2014; Edwards et al., 2020; Schlosser-Brandenburg et al., 2021; Yang et al., 2022; Barfod et al., 2023). Importantly, secondary microbial colonization can partially restore alveolar density and expand the gas exchange surface (Yun et al., 2014), highlighting that early-life microbial exposure may not only modulate signaling pathways but could also actively contribute to the dynamic maturation of the lung architecture.

Aromatic hydrocarbons, as well as tryptophan derivatives, are ligands for the aryl hydrocarbon receptor (AhR), which can modulate TGF-β signaling (Zimmerman et al., 2024; Hagan et al., 2025). SCFA, including butyrate, can modulate gene expression by inhibiting histone deacetylases (HDAC), an epigenetic mechanism that may influence key developmental signaling pathways, including BMP and FGF (Hinnebusch et al., 2002; Herriges and Morrisey, 2014).

In parallel, microbial modulation of the gamma-aminobutyric acid (GABA) system may also contribute to lung organogenesis. GABA_A_ receptors, which regulate chloride ion fluxes that are essential for fetal lung fluid secretion and airway distension, are critical for normal lung development, as demonstrated in murine models (Chintagari et al., 2010; Saito et al., 2010). Certain microbial metabolites have been shown to interact with this pathway, e.g. butyrate can increase GABA_A_ receptor gene expression via HDAC inhibition (Bohnsack et al., 2018), 5-aminovaleric acid betaine, a structural analogue of GABA, may function as a partial agonist (Luzzi et al., 1985) and polyamines like spermine, spermidine and putrescine may modulate the function of GABA_A_ receptors (Gilad et al., 1992; Limon et al., 2019; Kovács et al., 2021). Moreover, under certain conditions, putrescine may serve as substrate for GABA synthesis through an alternate pathway (Yoon and Lee, 2014; Kovács et al., 2021). Interestingly, these polyamines are also dynamically enriched in the embryonic chick lung during development, suggesting a potential time- and organ-specific role in lung morphogenesis, possibly through modulation of ion channel activity (Löwkvist et al., 1985).

#### 3.3.2 Immune system regulation

The microbiome plays an important role in immune education by shaping immune responses and promoting tolerance to environmental antigens and allergens. At birth, the neonatal immune system is naturally biased towards a Th2 phenotype, with limited Th1 responses and incomplete regulatory mechanisms (Zaghouani et al., 2009; Basha et al., 2014). This immune immaturity contributes to both an increased susceptibility to infections and an intrinsic propensity for dysregulated (such as allergic) inflammation (Zhang et al., 2017). Over time, probably within the six first months of life, the microbiome drives progressive immune maturation to establish balanced Th1/Th2 and regulatory responses.

In the respiratory tract, immune cells patrol the mucosal surface, which is constantly exposed to environmental antigens while preventing excessive responses to harmless stimuli. This high level of tolerance is largely ensured by contributions from, and interactions between, surface epithelium and myeloid cells including dendritic cells and alveolar macrophages. Regulatory cells exert immunomodulation through the induction of regulatory T cells (T_reg_) and the release of mediators such as prostaglandin E2, TGF-β, and interleukin (IL)-10 (Soroosh et al., 2013; Hussell and Bell, 2014). The two types of antigen-presenting cells (APC), together with epithelial cells, express pattern recognition receptors (PRR) such as Toll-like receptors (TLR), NOD-like receptors (NLR), C-type lectin receptors (CLR), retinoic acid-inducible gene-I-like receptors (RLR) and protease-activated receptors (PAR). Once activated, PRR induce the production of inflammatory cytokines, type I interferons, antimicrobial peptides and promote leukocyte recruitment (Chen et al., 2025). However, this system must ultimately discriminate between “danger” and “safe” signals, even though many PRR ligands are shared between commensals and pathogens (Chu and Mazmanian, 2013). This discrimination is achieved through subtle mechanisms such as compartmentalized PRR expression (Dickson et al., 2025), minor structural differences in microbe-associated molecular patterns (Lebeer et al., 2010), physical exclusion of commensals from epithelial contact by the mucus layer and, importantly, by the secretory IgA (S-IgA) system. Some pathogens, equipped with virulence factors and/or present in large abundance, can breach these barriers and trigger inflammation, whereas commensals generally remain shielded. A controlled level of PRR activation by commensal-derived signals is not only tolerated but essential for homeostasis, as shown in gut studies using *in vitro* and mice models. These studies demonstrated that microbial metabolites such as indole, together with mucosal IgA and antimicrobial peptides, promote epithelial barrier integrity by reinforcing tight junctional and cytoskeletal proteins and by fostering the recruitment of tolerogenic immune cell subsets, including T_reg_ and APC (Peterson et al., 2007; Bansal et al., 2010; Hapfelmeier et al., 2010).

The influence of microbiome on host homeostasis likely begins before birth. Notably, the polymeric immunoglobulin receptor (pIgR), a key component of the mucosal immune system (see IgA biology, section 4), is already widely expressed in the respiratory tract as early as the 4^th^ week of embryonic development (Ben-Hur et al., 2004), well before the appearance of a functional B cell compartment. Recently, studies in germ-free mice demonstrated that certain metabolites, including the Glu-Trp dipeptide, correlate with decreased gut *pIgR* gene expression *in utero* (Husso et al., 2023). In addition, studies in the gut showed that pIgR expression can be regulated by the microbiota, potentially through TLR- and MyD88-dependent pathways (Johansen and Kaetzel, 2011). SCFA also exert direct effects on the fetal lung, notably by promoting the induction of T_reg_ via G protein-coupled receptors (GPCR) signaling and direct inhibition of HDAC (Al Nabhani and Eberl, 2020). Moreover, bacterial DNA which is detected *in utero*, contains unmethylated cytosine-phosphate-guanine (CpG) oligodeoxynucleotide motifs (Satokari et al., 2009) that can bind TLR9 and induce Th1 and T_reg_ responses that can counterbalance the neonatal Th2 bias (Gupta and Agrawal, 2010). Together, these findings support the concept that both microbial DNA and microbiota-derived metabolites shape mucosal immune development during fetal life.

#### 3.3.3 Defense against pathogens

After birth, continuous exposure to microbial components such as lipopolysaccharide (LPS) further shapes lung immunity. The lipid A domain of LPS is recognized by TLR4, triggering a signaling cascade via the NF-κB pathway and leading to the production of pro-inflammatory cytokines such as IL-1β, IL-6, IL-8/CXCL8, IFN, and TNF-α (Luo et al., 2025). Studies in germ-free neonatal mice have shown that the absence of microbial colonization leads to exaggerated inflammatory responses to allergens, which are alleviated once the lower respiratory tract becomes colonized (Herbst et al., 2011). Another murine model showed that exposure to LPS or farm dust extract could prevent the development of allergen-driven asthma by suppressing epithelial and dendritic cell activation through induction of the ubiquitin-modifying enzyme A20 (Schuijs et al., 2015), providing a mechanism for the reduced prevalence of atopic diseases in children growing in ancestral farms as compared to their counterparts from close but ‘industrialized’ cities. Consistently, there is evidence that the lung microbiota contributes to the establishment of an immune-tolerant microenvironment, although the underlying mechanisms remain incompletely understood. In mice, the presence of microbiota may downregulate TLR4 expression, attenuate LPS-induced inflammation, and dampen the innate immune response of alveolar macrophages (Marquant et al., 2021). Early after birth, the bacterial load in the lung increases and the microbial composition shifts, which is associated with a weakened response to aeroallergens and PD-L1–driven emergence of T_reg_ (Gollwitzer et al., 2014). In healthy adults, the presence of commensals (such as *Prevotella* and *Veillonella*) in BAL has been associated with increased Th17 and neutrophilic responses compared to individuals without those detectable commensals, highlighting the immunoregulatory potential of these microbes (Segal et al., 2016). This concept is further supported by a murine study showing that intratracheal application of oral commensals (i.e. mixture of *Prevotella melaninogenica*, *Veillonella parvula* and *Streptococcus mitis*) can protect mice against *Streptococcus pneumoniae* infection, via the induction of a MyD88-dependent protective Th17 response (Wu et al., 2021).

Altogether, microbial signals educate the lung immune system by contributing to its regulation, promoting immune tolerance to allergens and supporting the maturation and long-term maintenance of immune homeostasis.

The lung microbiome also likely plays a substantial role in defending against pathogens. This protection may arise through both direct and indirect interactions within microbial communities and across kingdoms (Mac Aogáin et al., 2021), including competition and exchange of nutrients and oxygen, production of antimicrobial or toxic metabolites, physical interactions, quorum sensing, and modulation of biofilm formation (Pérez-Cobas et al., 2023b). These interactions can be synergistic or antagonistic, shaping community composition and function and contributing to resistance against pathogen invasion. While such interactions prevail in healthy lungs, dysbiosis in disease states may facilitate immune evasion and antimicrobial tolerance, selectively favoring the expansion of opportunistic species under specific microenvironmental conditions (Dickson et al., 2014b). For instance, eukaryotic viruses depend on host cellular machinery for replication, phages can reshape bacterial populations through lysogenic conversion, and methanogenic archaea engage in symbiotic metabolic cooperation with anaerobic bacteria by consuming metabolic by-products to produce methane, transferring hydrogen molecules or modulating environmental pH. Such cross-kingdom cooperation may enhance community efficiency and indirectly influence disease processes by altering microbiota structure (Kuehnast et al., 2025).

The modulation of the lung microbiota is not limited to local interkingdom crosstalk but also involves intersystem crosstalks, highlighting its systemic influence. A prototypic example is the gut–lung axis (GLA), which integrates anatomical, systemic, and nervous system connections mediating reciprocal exchanges of microbial signals between the lungs and the gut. Although mechanical processes such as gastroesophageal inhalation and sputum swallowing partially explain this connection, the GLA also relies on indirect pathways including translocation of microbial content or metabolites and cytokines through the bloodstream and lymphatics, as well as the migration of immune cells between both organs (Bingula et al., 2017; Nagpal and Yadav, 2017). For example, bacteria taken up in the gut by dendritic cells or macrophages can prime naïve B and T cells that subsequently migrate to the lungs (Bingula et al., 2017). The bidirectional nature of the GLA is now well recognized. Gut dysbiosis has been linked to an increased risk of respiratory infections, asthma, and the development or exacerbation of chronic pulmonary diseases (Abrahamsson et al., 2014). For instance, dietary modifications in newborns influence the lung microbiota (Madan et al., 2012a), and fecal transplantation in mice alters lung microbial communities (Liu et al., 2017; Li et al., 2021). Conversely, lung inflammation can induce systemic changes that affect gut composition. For example, intratracheal LPS instillation disrupts intestinal microbiota (Sze et al., 2014). Influenza lung infection in mice causes gut dysbiosis (Looft and Allen, 2012) and intestinal injury, which is mediated by T-cells migrating from the respiratory tract to the intestinal mucosa (Wang et al., 2014).

Beyond the gut, emerging evidence also points to a brain–lung axis (Huang et al., 2025), although current knowledge remains largely based on animal studies. Brain-to-lung sympathetic signaling can induce interstitial macrophage–mediated inflammation and lung injury (Li et al., 2025), while inhibition of this pathway attenuates cytokine storms and improves survival in rat models of severe pneumonia.

Conversely, lung microbiota dysbiosis may contribute to autoimmune neurological diseases, with strong associations reported between lung dysbiosis and multiple sclerosis in experimental models (Hosang et al., 2022; Alskaf, 2023). In rat models of experimental autoimmune encephalomyelitis, immune cells are activated within the lung before migrating to the central nervous system, highlighting the lung as a site of immunological priming preceding CNS infiltration (Odoardi et al., 2012). Evidence in humans, however, remains very limited, underscoring the need for further translational research. Analysis of autopsied brain tissue from multiple sclerosis patients have revealed the presence of bacterial species and phages (Branton et al., 2016). As these bacteria resemble environmental bacteria, lung epithelium has been proposed as potential route to CNS entry for microbial translocation.

Mechanistically, the lung microbiome appears to continuously transmit immunomodulatory signals to immune cells in the brain, favoring type I interferon pathways and thereby influencing susceptibility to autoimmune diseases (Hosang et al., 2022). Pulmonary dysbiosis, particularly altered LPS levels, has also been linked to oxidative stress, neuroinflammatory cascades, and neuronal apoptosis (Wu et al., 2025). Notably, its modulation in rat models of repetitive closed-head injury demonstrated neuroprotective effects (Wu et al., 2025), further highlighting the bidirectional and functional nature of the brain–lung axis.

## 4 Interactions between the lung microbiome and secretory IgA

**Figure 3:**
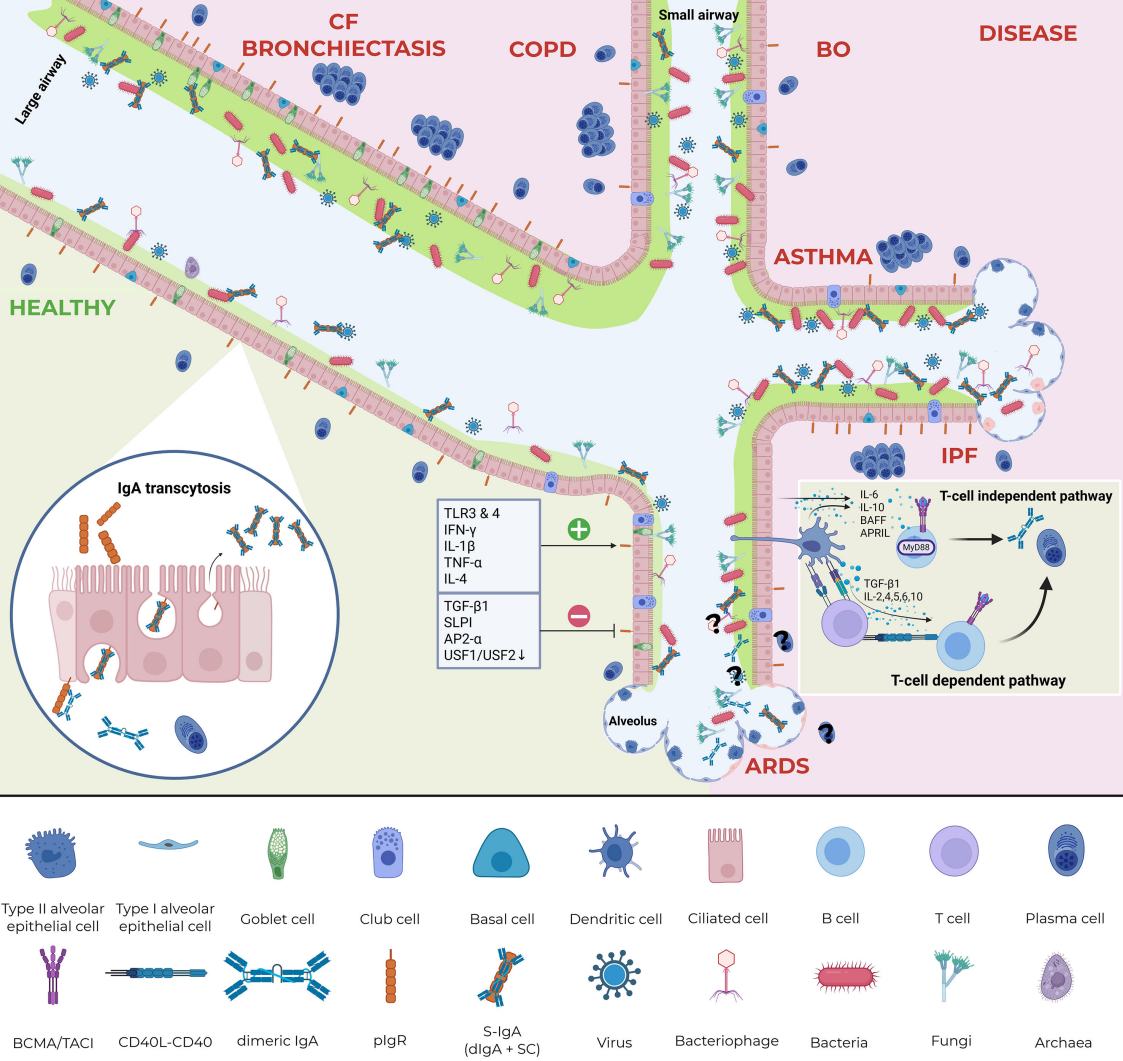
Dysregulation of the IgA–pIgR axis and microbiota at the mucosal interface. Green areas depict the healthy lung environment with intact IgA transcytosis in which dimeric IgA (dIgA) produced by B cells through T-dependent or T-independent pathways is transported across the epithelium via the polymeric immunoglobulin receptor (pIgR) and released into the airway lumen as secretory IgA (S-IgA). Transcytosis of unbound pIgR results in the release of free secretory component (SC). Several factors can positively or negatively regulate pIgR expression. In addition, the red area illustrates the disease lung environment, showing alterations in the airway microbiota, reduced or aberrant (ectopic) pIgR expression, and altered levels of S-IgA in cystic fibrosis (CF), bronchiectasis, chronic obstructive pulmonary disease (COPD), bronchiolitis obliterans (BO), idiopathic pulmonary fibrosis (IPF) or acute respiratory distress syndrome (ARDS). Lymphoid follicles are also illustrated, and insets show pIgR-mediated transport of d-IgA (left) and pathways involved in IgA synthesis by B cells (right). Created with Biorender.

IgA represents the major protective antibody isotype at mucosal surfaces. Although IgA circulating in serum is predominantly monomeric, mucosal IgA is primarily synthesized in a dimeric form (d-IgA) by local subepithelial plasma cells (Woof and Kerr, 2006). Dimeric-IgA consists of two IgA monomers, covalently linked by a joining peptide (J chain), which facilitates its binding to the pIgR expressed on the basolateral surface of epithelial cells. Two subclasses of IgA exist in humans, i.e. IgA1 and IgA2. IgA1 has a longer, pathogen-derived protease-sensitive hinge region and predominates in serum, whereas IgA2, with a shorter and more resistant hinge, is relatively enriched in mucosal secretions (Woof and Kerr, 2006). Upon binding, the d-IgA/pIgR complex undergoes clathrin-mediated endocytosis and transcytosis across the epithelium (**Figure 3**). At the apical pole, a proteolytic cleavage of pIgR releases secretory IgA (S-IgA), composed of d-IgA and the extracellular fragment of pIgR, known as the secretory component (SC). Transcytosis also occurs without the binding of d-IgA and provides free SC in mucosal secretions.

The S-IgA system mediates so-called immune exclusion of pathogens and toxins, preventing their adhesion to the surface epithelium, neutralizing them within the epithelial layer, as well as regulating leukocyte responses (de Fays et al., 2022). In addition to its defensive role, S-IgA also contributes to the regulation of microbiota composition (Bunker and Bendelac, 2018). SC itself may protect S-IgA from proteolytic degradation, enhance its binding to mucus and bacterial surfaces, and increase its capacity to neutralize viruses. Moreover, SC possesses intrinsic antimicrobial and anti-inflammatory properties, further contributing to mucosal barrier homeostasis (de Fays et al., 2022).

Given the central role of IgA in mucosal immunity, understanding the origin and regulation of B cells responsible for its production is essential, as reviewed by our team (Sánchez Montalvo et al., 2022; Bertrand et al., 2023). Briefly, conventional B cells (B_2_ cells) originate from common lymphoid progenitors in the bone marrow. Their early development is driven by cytokines such as IL-7, and supported by the CXCL12–CXCR4 chemokine axis, which retains immature precursors within the bone marrow niche and guides their progression through successive stages of Ig gene rearrangement (Clark et al., 2014). Throughout its maturation, B cells exit the bone marrow and complete their development in peripheral lymphoid tissues. Their survival and functional maturation depend on interactions with B cell activating factor (BAFF) and a proliferation-inducing ligand (APRIL). Following antigen exposure, naïve B cells are activated in secondary lymphoid organs, within germinal centers, through cognate interactions with Th cells. This T cell–dependent pathway promotes somatic hypermutation, class switch recombination (CSR), and the generation of high-affinity memory B cells and long-lived plasma cells. The CSR toward IgA is predominantly driven by low concentration of TGF-β1 and CD40–CD40L interactions. Other cytokines, including IL-2, IL-4, IL-5, IL-6, and IL-10, can synergize with CD40L by stimulating TGF-β1 production in B cells, thereby creating an autocrine loop that drives IgA synthesis. In addition to this T cell–dependent pathway, IgA CSR can also occur through T cell–independent mechanisms, especially in mucosal tissues.

### 4.1 Ontogeny of lung mucosal IgA

Serum and airway mucosal IgA, which are extremely low at birth due to both lack of placental transfer and immaturity of the adaptive immune system, rise slowly throughout infancy and early childhood, to reach adult concentrations around puberty (Buckley et al., 1968; Burgio et al., 1980; Jolliff et al., 1982; Forsyth et al., 1988; Irjala et al., 1990; Sennhauser et al., 1990; Weemaes et al., 2003). In contrast, SC is already detectable in saliva and shows no significant age-related variation (Burgio et al., 1980). This, together with the early *in utero* expression of pIgR in the respiratory tract (Ben-Hur et al., 2004), may indicate that the epithelial transcytosis machinery is already functional early in life, while the interaction with IgA and the production of IgA itself still require further maturation. Thus, the earliest signs of the B cell lineage are detectable around 7 to 9 weeks gestational age (Popescu et al., 2019; Park et al., 2020) with functional Ig synthesis only starting around 16 weeks of gestation (Rechavi et al., 2015). From this stage onward, both the number and the repertoire of fetal B cells progressively expand *in utero* (Rechavi et al., 2015; Park et al., 2020). However, germinal center formation and CSR remain limited until after birth (Burgio et al., 1980; Jolliff et al., 1982). This reflects the immaturity of both T-dependent and B cell–intrinsic mechanisms, with reduced CD40L expression on T cells, lower expression of BAFF and APRIL receptors, and weak IgA responses to CD40L and IL-10 stimulation (Rechavi et al., 2015).

In the lung specifically, B cell recruitment at birth is initially sparse and mainly composed of naïve B cells but progressively increases and differentiates under the influence of microbial colonization and local immune signaling from epithelial cells, DC and alveolar macrophages (Hoytema van Konijnenburg et al., 2024). Consistent with this, early airway microbial colonization is associated with the upregulation of both host IgA-related and microbial anti-IgA pathways, reflecting a microbiota-driven priming of mucosal immunity and early host–microbe crosstalk (Pattaroni et al., 2018). However, these IgA antibodies show low affinity and specificity, as evidenced around 5 months of age by somatic mutation frequencies of only ∼25% of adult values and poor antigen-driven selection (Rogosch et al., 2012). Additional evidence suggests that serum and mucosal IgA are not derived from a single uniform B cell pool, but rather from distinct B cell compartments, with some clones contributing predominantly to systemic IgA and others to mucosal IgA (Aihara et al., 2023). This separation likely contributes to the differential kinetics and functions of IgA in blood and mucosal sites.

### 4.2 Microbial-specific IgA responses

IgA specificity arises from the interplay between T-dependent and T-independent pathways. Because T-cell dependent CSR toward IgA takes at least 5 to 7 days (Cerutti, 2008), rapid protection is ensured by T-independent mechanisms that generate low-affinity, polyreactive IgA. This can occur through direct activation of B cells – e.g. (via TLR4) by LPS or polysaccharides, or indirectly via cytokines such as BAFF and APRIL produced by dendritic, epithelial, or stromal cells, notably through activation of the MyD88 signaling pathway (Bunker and Bendelac, 2018). Together, polyreactive and high-affinity IgA antibodies shape mucosal immunity and regulate host–microbiota interactions. Supporting this concept, transient gut colonization in germ-free mice is sufficient to induce microbiota-specific S-IgA (Hapfelmeier et al., 2010), highlighting dynamic adaptation to o the nature, intensity, and duration of microbial exposure.

In the gut, this adaptation is also reflected in the distribution of IgA isotypes along the intestinal axis, forming a concentration gradient (with progressive enrichment in IgA2 vs IgA1) from proximal to distal gut and from the mucosal epithelium toward the lumen (Tejedor Vaquero et al., 2024). About half of the bacteria in the ileum are coated by IgA, compared to only ∼10% in the colon (Sterlin et al., 2019). Both subclasses targeting overlapping microbes, dual IgA1 and IgA2 coating is considered as a hallmark of intestinal homeostasis, but some commensals can also be selectively bound by IgA2 (Sterlin et al., 2019; Tejedor Vaquero et al., 2024). In general, CSR to IgA2 occurs more frequently via T cell–independent pathways, whereas IgA1 switching may rather depend on T-cell interactions (Sterlin et al., 2019). In inflammatory bowel disease, this balance is disrupted (Shapiro et al., 2021), with expansion of IgA1-producing B cells and depletion of IgA2-producing B cells (Sterlin et al., 2019). This shift enhances IgA1 reactivity toward pathobionts but compromises protection toward commensals, suggesting a transition toward a more pro-inflammatory IgA1/IgG profile.

The exact proportions and specific functions of each IgA isotype in lung homeostasis and host defense are much less characterized than in the gut. While individuals with selective IgA deficiency may exhibit gut microbiota dysbiosis (Fadlallah et al., 2018; Catanzaro et al., 2019), a small cohort study suggested that IgA is not essential for maintaining a functional salivary microbiome (de la Cruz Peña et al., 2021). This highlights the site-specific contribution of IgA to mucosal homeostasis and raises the question of whether similar principles apply to the respiratory tract.

The overall efficiency of this system further depends on IgA binding site and on the regulation of pIgR expression, which governs IgA transcytosis and generation of S-IgA. IgA can bind antigens either through its variable region, shaped by somatic hypermutation, or via noncanonical regions, particularly its heavily glycosylated portions (van de Bovenkamp et al., 2018). The structure and abundance of these glycans vary considerably between individuals, mucosal sites, and inflammatory states, as well as with the rate of IgA production. Beyond providing a structural framework for antigen interactions, these glycans can serve as nutrient sources (Briliūtė et al., 2019) and act as signaling molecules that modulate bacterial gene expression (Nakajima et al., 2018), thereby shaping the composition and behavior of the microbiota. Proinflammatory cytokines such as IFN-γ, IL-1β, TNF-α, and IL-4, as well as TLR3 or TLR4 activation, upregulate *PIGR* transcription via distinct pathways, including IRF-1, NF-κB, and STAT6 (Blanch et al., 1999; Schjerven et al., 2000). Notably, some pathways, such as TLR3, elicit a more pronounced inflammatory response than others, like TLR4, which may reflect a mechanism distinguishing activation by pathogens versus commensal microbes (Schneeman et al., 2005). In contrast, TGF-β1, secretory leukocyte protease inhibitor (SLPI), and altered expression of transcription factors such as upstream stimulatory factor (USF) 1 or 2 or Activator Protein (AP)2-α are associated with downregulation of *PIGR* expression (Khattar et al., 2005; Gohy et al., 2014; Mikami et al., 2015). This regulatory balance determines the amount of S-IgA available at mucosal surfaces, thereby influencing the shaping of microbiota by host immunity.

## 5 Alterations in anti-microbial IgA responses in lung disease

Despite their heterogeneous etiologies and pathophysiological mechanisms, several lung diseases share a defect in S-IgA–mediated immune defense (de Fays et al., 2022). Whether this mucosal alteration connects with shifts in the respiratory microbiome (summarized in **Table 1**) and with dynamic changes in host-microbial interplays, will be discussed according to evidence in each major lung disorder (**Figure 3**).

**Table 1:** Overview of alterations in mucosal IgA–epithelium–microbiota landscapes in major lung diseases.

Disease		Mucosal/airway IgA					Airway microbiota		
		*In vivo (in situ)*			*In vitro* (epithelium)		Bacteriota	Virota	Mycobiota
		S-IgA	pIgR	IgA+ B cells	S-IgA	pIgR			
CF		↑ (Konstan et al., 1994; Collin et al., 2020)	↑ (Marshall et al., 2004; Collin et al., 2020)	↑ (Martinez-Tello et al., 1968; Frija-Masson et al., 2017; Collin et al., 2020)	↓ (Collin et al., 2020)	↓ (Collin et al., 2020)	Pseudomonas↑ Haemophilus↑ Burkholderia ↑ Streptococcus ↑ Staphylococcus ↑ Stenotrophomonas ↑ (Moran Losada et al., 2016; Cuthbertson et al., 2020; Cauwenberghs et al., 2024; Motta et al., 2024)	Bacteriophage ↑ Herpesvirus ↑ Adenovirus ↑ (Moran Losada et al., 2016)	Candida ↑ Aspergillus ↑ Scedosporium ↑ Exophilia ↑ Saccharomyces ↑ Malassezia ↑ (Moran Losada et al., 2016; Cuthbertson et al., 2021; Hong et al., 2023; Angebault and Botterel, 2024)
Bronchiectasis		↑ (Waite et al., 1983; Burnett et al., 1987; Plusa and Wäsek, 1987; Burnett et al., 1990; Vasil’ev et al., 1996; Atiş et al., 2001)	NA	↑ (Whitwell, 1952; Silva et al., 1989; Frija-Masson et al., 2017)	NA	NA	Streptococcus ↑ Haemophilus ↑ Pseudomonas ↑ (Richardson et al., 2019; Mac Aogáin et al., 2021; Motta et al., 2024; Wang et al., 2025)	Bacteriophage ↑ Parainfluenza ↑ Rhinovirus ↑ Influenza A↑ (Mac Aogáin et al., 2021; Wang et al., 2025)	Candida ↑ Saccharomyces ↑ Aspergillus ↑ Penicillium ↑ (Mac Aogáin et al., 2021; Wang et al., 2025)
COPD		↓ (Medici and Buergi, 1971; Deuschl and Johansson, 1974; Soutar, 1977; Stockley et al., 1981; Demoly et al., 1999; Polosukhin et al., 2011; Du et al., 2015; Polosukhin et al., 2017; Liu et al., 2020; Southworth et al., 2021; Di Stefano et al., 2022)	↓ (Medici and Buergi, 1971; Polosukhin et al., 2011; Gohy et al., 2014; Du et al., 2015)	↑ (Hogg et al., 2004; Polosukhin et al., 2011; Ladjemi et al., 2015; Ladjemi et al., 2019; de Fays et al., 2023)	↓ (Gohy et al., 2014)	↓ (Gohy et al., 2014; Carlier et al., 2024)	Streptococcus ↑ Pseudomonas ↑ Haemophilus ↑ Moraxella ↑ Actinomyces ↑ Neisseria ↑ Prevotella ↓ (Einarsson et al., 2016; Wang et al., 2020; Ramsheh et al., 2021; Sulaiman et al., 2023; KavianFar et al., 2025)	Bacteriophage ↓ Anelloviruses ↑ (Garcia-Nuñez et al., 2018; Van Rijn et al., 2019; Cook et al., 2025)	Candida ↑ Aspergillus ↑ Cladosporium ↑ Malassezia ↑ (Liu et al., 2021; Martinsen et al., 2021; Tiew et al., 2021; Wang et al., 2023)
Asthma	T_2_ ↑	=/↑ (Salvaggio et al., 1973; Plusa and Wäsek, 1987; del Castillo Aguas et al., 1988; Van De Graaf et al., 1991; Stokes Peebles et al., 1995; Van Vyve et al., 1995; Louis et al., 1997; Nahm and Park, 1997; Nahm et al., 1998; Demoly et al., 1999; Fouda, 2004; Balzar et al., 2006; Adel-Patient et al., 2021)	↓ (Ladjemi et al., 2018)	NA	= (Ladjemi et al., 2018)	= (Ladjemi et al., 2018)	Streptococcus ↑ Tropheryma ↑ Actinomyces ↑ (Simpson et al., 2016; Zhang et al., 2016; Li et al., 2017; Taylor et al., 2018)	Bacteriophage ↓ Picornaviruses ↑ Anelloviruses ↑ Herpesviridae ↑ (Choi et al., 2021; Megremis et al., 2023)	Fusarium ↑ Cladosporium ↑ Aspergillus ↑ Alternaria ↑ (Sharma et al., 2019; Vandenborght et al., 2021; Yang et al., 2022)
	T_2_ ↓						Moraxella ↑ Neisseria ↑ Haemophilus ↑ (Sverrild et al., 2017; Yang et al., 2018; Durack et al., 2020)		Mycosphaerella ↑ Basidiomycota ↑ (Sharma et al., 2019; Vandenborght et al., 2021; Yang et al., 2022)
**BO**		↓ (Bastian et al., 2000; Carlier et al., 2025)	↓ (Carlier et al., 2025)	= (Carlier et al., 2025)	NA	NA	Pseudomonas ↑ (except CF post LTx) Veillonella ↑ Streptococcus ↑ Staphylococcus ↑ Corynebacterium ↑ Propionibacterium ↑ (Botha et al., 2008; Willner et al., 2013; Schott et al., 2018; Metwally et al., 2020; Combs et al., 2021; Martin et al., 2024)	CMV ↑ Anellovirus ↑ (Weigt et al., 2008; Young et al., 2015; Schott et al., 2018)	Aspergillus ↑ (Weigt et al., 2009; Willner et al., 2013)
**IPF**		↑ (Mills et al., 2019; Kobayashi et al., 2020; Boustani et al., 2022; Planté-Bordeneuve et al., 2024)	↑/ectopic (Planté-Bordeneuve et al., 2024)	↑ (Heukels et al., 2019)	NA	NA	Haemophilus ↑ Streptococcus ↑ Neisseria ↑ Veillonella ↑ Bacteroides ↓ (Molyneaux et al., 2014; Tong et al., 2019; Knudsen et al., 2025)	Bacteriophage ↑ Herpesvirus ↑ (Tong et al., 2019; Sheng et al., 2020)	Candida ↑ Aspergillus ↑ Malassezia ↑ (Molyneaux et al., 2016; Roudbary et al., 2019)
**ARDS**		= (Gerard et al., 2024)	↓ (Gerard et al., 2024)	NA	NA	NA	Bacteroides ↑ Enterobacteriaceaea ↑ Staphylococcus ↑ Ralstonia ↑ Enterococcus ↑ (Dickson et al., 2016; Panzer et al., 2018; Dickson et al., 2020; Montassier et al., 2023)	Herpesvirus ↑ (Mitsuyama et al., 2025)	Candida ↑ Aspergillus ↑ (Viciani et al., 2022; Britton et al., 2023)

S-IgA, secretory immunoglobulin A; pIgR, polymeric immunoglobulin receptor; CF, cystic fibrosis; COPD, chronic obstructive pulmonary disease; BO, bronchiolitis obliterans; ARDS, acute respiratory distress syndrome; LTx, Lung transplantation. NA, data not available.

### 5.1 CF and non-CF bronchiectasis

CF and non-CF bronchiectasis share a remarkably similar airway microbiome dysbiosis, with enrichment in *Proteobacteria* (*Pseudomonas*, *Haemophilus*) and *Firmicutes* (*Streptococcus*) (Moran Losada et al., 2016; Richardson et al., 2019; Cuthbertson et al., 2020; Mac Aogáin et al., 2021; Cauwenberghs et al., 2024; Motta et al., 2024; Wang et al., 2025), reflecting a shift toward an increased representation of pathogenic facultative and strict anaerobes accompanied by a reduction in the relative abundance of anaerobic commensals. In parallel, elevated loads of phages and fungi (*Candida*, *Aspergillus, Saccharomyces*) have been reported as compared to healthy controls (Moran Losada et al., 2016; Richardson et al., 2019; Mac Aogáin et al., 2021; Angebault and Botterel, 2024; Wang et al., 2025). These microbial community changes reflect adaptation to a complex, inflamed airway environment that is rich in mucus and relatively oxygen-depleted. Such conditions favor biofilm formation and microbial phenotypic and genotypic adaptation, particularly by *Pseudomonas aeruginosa (Pa)*. Pa also tolerates and can even thrive in such conditions, which promote its growth and persistence (Sánchez-Clemente et al., 2018) notably through the mucus properties, rich in carbon and energy sources such as alanine and lactate (Palmer et al., 2007). Similarly, *Candida* is well adapted to survive in oxygen-poor environments (Setiadi et al., 2006).

These microbial alterations are accompanied by a unique (as compared to other chronic lung diseases described below) and pronounced activation of the mucosal S-IgA system, characterized by marked increases in both IgA levels in airway secretions (Clarke, 1976; Turnbull et al., 1978; Waite et al., 1983; Burnett et al., 1987; Plusa and Wäsek, 1987; Burnett et al., 1990; Konstan et al., 1994; Vasil’ev et al., 1996; Atiş et al., 2001; Collin et al., 2020; Mottais et al., 2025) and pIgR expression in the airway epithelium (Collin et al., 2020), along subepithelial lymphoid aggregates (Whitwell, 1952; Silva et al., 1989; Hubeau et al., 2002; Frija-Masson et al., 2017; Polverino et al., 2019) and expansion of IgA-producing B cells (Martinez-Tello et al., 1968; Collin et al., 2020). The underlying mechanisms, which may include IL-17–mediated pathways (Collin et al., 2020), remain largely elusive at this stage. In CF, SC production is also increased, while its proteolytic degradation and altered glycosylation prevent its ability to bind IL-8/CXCL8, subsequently promoting neutrophilic airway inflammation (Marshall et al., 2004). The increase in S-IgA in CF lungs was correlated with chronic bacterial infection, especially Pa (Collin et al., 2020). In non-CF bronchiectasis, this increase appears to be selectively associated with Pa colonization and is not observed in patients colonized by other pathogens (*Haemophilus influenzae*, *Streptococcus pneumoniae*, or *Klebsiella pneumoniae*) (Noda et al., 1992). Indeed, several previous studies showed an enrichment of IgA antibodies against Pa (Clarke, 1976; Mottais et al., 2025), predominantly targeting LPS, especially lipid A and O polysaccharide epitopes rather than the core region (Clarke, 1976; Kronborg et al., 1992). IgA reactivity was also observed against cytoplasmic antigens of *Haemophilus influenzae* without correlation to culture positivity, while no specific responses were detected against *Streptococcus pneumoniae*, *Klebsiella pneumoniae* or *Aspergillus fumigatus* in a small cohort study, with a similar pattern observed in additional patients with bronchiectasis (Clarke, 1976). More recently, IgA targeting staphylococcal enterotoxin B has been shown to be elevated (Mottais et al., 2025), whereas specificities against other microbiota members remain unknown.

Emerging evidence also indicates the presence of serum IgA directed against LPS of respiratory pathogens, including Pa and *Burkholderia* species (Pham et al., 2021; Pham et al., 2024). Paradoxically, these specific IgA inhibit complement-mediated killing in a titer- and epitope-specific affinity-dependent manner. In Pa, O-antigen–specific IgA, -but not IgA against the common polysaccharide antigen, another surface-exposed LPS component-impair complement-mediated killing (Monteith et al., 2026). These observations are mainly derived from *ex vivo* assays using sera from chronically infected individuals incubated with clinical sputum isolates, highlighting the functional impact of circulating antibodies on bacterial survival. Importantly, depletion of O-antigen–specific IgA by plasmapheresis restores serum bactericidal activity and can lead to the clearance of Pa in sputum (Divithotawela et al., 2020), underscoring a role for serum IgA in shaping airway colonization. Together, these observations emphasize that IgA binding does not automatically confer effective host defense and, in some contexts, may even represent a maladaptive immune response that interferes with other antibacterial mechanisms.

Interestingly, one study assessing IgA subclasses showed a selective rise in IgA1 but not IgA2 (Mottais et al., 2025), potentially linked to either a selective (T-cell dependent) IgA1 production or enhanced sialylation of IgA1, which could reduce its susceptibility to proteolytic degradation (Ohyama et al., 2020). In addition, IgA -but not IgG - autoantibodies against Bactericidal permeability-increasing protein have been detected in BAL, where they closely correlate with anti-*Pa* IgA levels (Theprungsirikul et al., 2020). Their presence in infected airways (and nor serum) of CF children could suggest a locally breached immune tolerance, while systemic IgG autoantibodies may arise later probably through distinct mechanisms. In contrast, anti-dsDNA IgA autoantibodies were significantly elevated in both serum and sputum, even in very young CF patients (Yadav et al., 2020), with serum (but not sputum) levels correlating with airway obstruction. These observations highlight that the nature of isotype responses differs between compartments, illustrating a dissociation between systemic and mucosal immunity, in addition to the B-cell compartmentalization that exists within the lung (see point 4.1). Although the revolutionary CFTR modulators treatment reduces systemic IgA levels (Schnell et al., 2023; Pepe et al., 2025), it does not appear to significantly affect local concentrations in the airways (Theprungsirikul et al., 2020; Mottais et al., 2025), possibly further emphasizing this compartment-specific regulation.

Finally, the relevance of boosted IgA responses in CF lungs remains largely uncertain, notably with regard to exacerbations or airway dysbiosis. The potential roles of defects in affinity maturation, antibody entrapment within thick mucus, or the presence of phenotypically diverse bacteria due to adaptation during the chronic disease process (Nickerson et al., 2024), could underlie this paradoxical association of increased IgA antibodies and propensity to lung dysbiosis and infections, and should deserve future research.

### 5.2 Asthma, COPD and bronchiolitis

Airway diseases like asthma, chronic obstructive pulmonary disease (COPD), and bronchiolitis obliterans (BO), while arising from distinct genetic and environmental factors, share several pathophysiological features that include airway inflammation and altered epithelium barrier.

In contrast with CF, these three diseases exhibit a downregulation of the S-IgA system at the epithelium pIgR level, whereas airway IgA levels may vary across studies. The reported variations in S-IgA (increased (Turnbull et al., 1978; Plusa and Wäsek, 1987; del Castillo Aguas et al., 1988; Van De Graaf et al., 1991; Louis et al., 1997; Nahm and Park, 1997; Nahm et al., 1998; Atiş et al., 2001), unaffected (Salvaggio et al., 1973; Stokes Peebles et al., 1995; Van Vyve et al., 1995; Nahm and Park, 1997; Demoly et al., 1999; Fouda, 2004; Balzar et al., 2006; Ohlmeier et al., 2012; Adel-Patient et al., 2021), or decreased (Medici and Buergi, 1971; Deuschl and Johansson, 1974; Clarke, 1976; Soutar, 1977; Stockley et al., 1981; Polosukhin et al., 2011; Polosukhin et al., 2017; Liu et al., 2020; Di Stefano et al., 2022)) may relate to differences in disease endotype (like T2-high vs T2-low), disease severity (Nahm and Park, 1997; Southworth et al., 2021) as well as to other factors. In COPD, these discrepancies may also relate to a relatively preserved pIgR expression (and thus S-IgA production) by submucosal glands (Du et al., 2015).

In “healthy” smokers and mild COPD, pIgR expression may remain close to levels in control non-smokers, but progressively reduces in remodeled bronchial epithelium in more advanced disease (Pilette et al., 2001; Gohy et al., 2014). *In vitro* models of airway epithelium reconstituted from COPD patients revealed that this defect is “imprinted” and maintained upon prolonged culture (Gohy et al., 2014; Carlier et al., 2024). Thus, in severe COPD, local S-IgA deficiency coincides with epithelium damage, airway remodeling/fibrosis, and accumulation of submucosal IgA-producing B cells (Burnett et al., 1987; Hogg et al., 2004; Polosukhin et al., 2011; Ladjemi et al., 2015; Polosukhin et al., 2017; Ladjemi et al., 2019; de Fays et al., 2023). These defects are particularly marked in distal airways and emphysematous regions and correlates with airflow limitation and disease severity (Pilette et al., 2001; Gohy et al., 2014).

In asthma, reduced epithelial pIgR expression has also been observed (Ladjemi et al., 2018), with variable levels of IgA. In addition, SC levels were also either decreased (Van Vyve et al., 1995), preserved (Louis et al., 1997) or increased (Marshall et al., 2004). reflecting differences in patient populations, assay methodologies or heterogeneity of airway alterations. Interestingly, a recent study demonstrated that anti–IL-5 therapy enhanced S-IgA production in mild asthma patients with low eosinophil counts (Sabogal Piñeros et al., 2019), suggesting that attenuating eosinophilic activation may promote mucosal IgA production. This therapy may also induce long-term improvements in epithelial integrity (Domvri et al., 2025), possibly promoting airway mucosal homeostasis.

In BO, where immune and fibroblast activation play central roles, reduced S-IgA levels (Bastian et al., 2000; Carlier et al., 2025) and pIgR expression (Carlier et al., 2025) have been reported while BAL S-IgA and serum SC could serve in the lung post-transplant setting as a biomarker to identify patients at risk of chronic rejection (Carlier et al., 2025).

Local S-IgA deficiency in lower airways may facilitate bacterial and viral infection or colonization (Polosukhin et al., 2011; Polosukhin et al., 2017). Thus, this immune defect parallels a characteristic airway dysbiosis. Changes in the disease airway microenvironment, such as increased mucus water content (Kelly et al., 2024), reduced oxygen tension, local acidification (Zajac et al., 2021), and sustained inflammation with redox imbalance, create a niche favorable to Gram-negative genera (*Pseudomonas, Haemophilus, Neisseria*), to mildly acidophilic genera (*Actinomycetes*, Firmicutes) (Duncan et al., 2009) as well as to facultative anaerobes like *Streptococcus* and *Moraxella*. Overrepresentation of these (potentially pathogenic) bacteria, as well as expansion of fungi (including *Aspergillus*), and a decreased of phages along increased *Anellovirus* abundance represent shared microbial signatures across chronic acquired/inflammatory airway diseases.

The airway microbiome composition, however, is far from being uniform and similarly to local IgA levels, varies according to disease endo/phenotype, atopic status, and treatment exposure (inhaled corticosteroids, antibiotics, bronchodilators, or biologic therapies) (George et al., 2019; Taylor et al., 2019; Leitao Filho et al., 2021; Huang et al., 2022). For instance, in T2-high asthma, bacteria such as *Strepcococcus*, *Tropheryma* and *Actinomyces* (Simpson et al., 2016; Zhang et al., 2016; Li et al., 2017; Taylor et al., 2018) and fungi such as *Fusarium*, *Cladosporium*, *Aspergillus*, and *Alternaria* are typically enriched (Sharma et al., 2019; Vandenborght et al., 2021; Yang et al., 2022), whereas T2-low asthma is characterized by *Neisseria, Moraxella, Haemophilus*, *Mycosphaerella* and Basidiomycota enrichment (including *Trametes, Papiliotrema, Trechisporales*) (Sverrild et al., 2017; Yang et al., 2018; Sharma et al., 2019; Durack et al., 2020; Vandenborght et al., 2021; Yang et al., 2022).

Together, these findings suggest a potential common pathogenic mechanism across acquired airway diseases, in which epithelium injury, altered mucosal immunity, and dysbiosis perpetuate each other in a self-reinforcing vicious cycle of inflammation and remodeling. However, the specific role of IgA in shaping the airway microbiome remains largely unexplored. In addition, only three studies examined IgA specificities. Early work detected IgA antibodies against *H. influenzae* (cytoplasmatic and cell wall antigens) in patients with asthma and chronic bronchitis, and against Pa (cytoplasmatic and cell wall antigens) in some bronchitis cases, while no reactivity was observed for *Klebsiella pneumoniae*, *Staphylococcus aureus*, *Streptococcus pneumoniae*, or *Aspergillus fumigatus* (Clarke, 1976). In allergic asthma, increased IgA response to *S. pneumoniae* has also been described (Nahm et al., 1998). More recently, in COPD, enhanced opsonizing IgA binding to *H. influenzae* was observed in eosinophil-high- vs low phenotype (Southworth et al., 2021), while specific Pa IgA levels remained low in severe COPD, slowly rising only when colonization occurred (Millares et al., 2017). This suggests that IgA production may be antigen-driven but not necessarily fully protective.

### 5.3 Parenchymal lung diseases

IPF and acute respiratory distress syndrome (ARDS) represent major (chronic or acute forms, respectively) alveolo-interstitial pathologies, underlined by different mucosal immune alterations. IPF is initiated by recurrent injury of alveolar type II (AT2) epithelial cells, which normally maintain surfactant metabolism and act as local progenitors for the alveolar epithelium. Under repeated stress, AT2 cells undergo maladaptive responses leading to progressive tissue fibrosis in the distal lung tissue (Koudstaal and Wijsenbeek, 2023). Associated mucosal alterations include increased S-IgA (Mills et al., 2019; Kobayashi et al., 2020; Boustani et al., 2022; Planté-Bordeneuve et al., 2024) and pIgR expression (Planté-Bordeneuve et al., 2024) as well as expansion of submucosal IgA-producing B cells (Heukels et al., 2019). Although IgA can exert both pro- and anti-fibrotic effects, *in vitro* studies suggest that S-IgA may promote profibrotic cytokine secretion by fibroblasts at both protein and mRNA levels, thereby potentially fueling disease progression (Arakawa et al., 2019).

In contrast, ARDS probably results from acute damage to AT1 cells and capillary endothelium, disrupting the alveolar-capillary barrier and increasing permeability (Thompson et al., 2017). Diffuse alveolar damage is accompanied by distal airway structural changes, including extensive epithelial denudation, dedifferentiation as well as decreased pIgR expression (Gerard et al., 2024). This leads to a local dysfunction of the IgA–pIgR system, with leakage of secretory proteins (S-IgA and SC) into the serum and translocation of non-secretory (dimeric) IgA from the circulation into the airway lumen (Gerard et al., 2024).

Both IPF and ARDS exhibit alterations of the airway microbiome, although the patterns and potential consequences may differ. In IPF, dysbiosis is characterized by increased abundance of *Haemophilus, Streptococcus, Veillonella*, and *Neisseria* and decreased abundance of *Bacteroides* (Molyneaux et al., 2014; Tong et al., 2019; Knudsen et al., 2025), as well as enrichment of herpesviruses (CMV, EBV, HHV-7, HHV-8) (Tong et al., 2019; Sheng et al., 2020) and fungi including *Candida*, *Aspergillus* and *Malassezia* (Molyneaux et al., 2016; Roudbary et al., 2019). This dysbiosis could trigger exaggerated immune responses, further amplifying epithelial injury and fibrotic remodeling. In ARDS, the airway microbiome remains poorly characterized. Available studies suggested that alterations are largely driven by gut-derived bacteria, with increases in *Bacteroides*, *Enterobacteriaceae*, *Staphylococcus*, *Ralstonia*, and *Enterococcus* (Dickson et al., 2016; Panzer et al., 2018; Dickson et al., 2020; Montassier et al., 2023). The airway mycobiome also showed a marked predominance of *Candida* (Viciani et al., 2022; Britton et al., 2023).

Importantly, the antigenic specificity and functional relevance of IgA responses in these diseases remains unknown. Some evidence suggested a potential contribution of IgA to local autoimmune processes (Solomon et al., 2020; Boustani et al., 2022), highlighting a critical gap in our understanding of the role of mucosal IgA immunity in the distal lung.

## 6 Scientific and therapeutic perspectives

The respiratory microbiome is a low biomass but highly dynamic ecosystem encompassing bacteria, viruses, fungi, and archaea, whose composition and activity likely shape mucosal structure, immune development and susceptibility to lung diseases. Within this complex multi-microbial ecosystem, interkingdom crosstalk and their reciprocal communication with host cells influence lung microbiome composition and function and may contribute to the pathogenesis of chronic diseases. The present review also highlights that several chronic lung diseases share similar pathogenic mechanisms and microbial signatures, suggesting common pathways of dysbiosis. A better understanding of these processes is essential to develop microbiome-targeted therapeutic strategies aimed at restoring both microbial and pulmonary homeostasis. Although still in their early stages, several approaches are being explored to modulate the respiratory microbiome. Prebiotics, probiotics, phages, and microbial metabolites represent promising approaches to selectively target pathogens and/or the overt host immune response, to restore a homeostatic microbial network. Most probiotic interventions have been developed for oral or intranasal administration, typically using commensal bacteria such as *Lactobacillus* and *Bifidobacterium*. Strain selection and dosage remain critical for therapeutic efficacy, yet no standardized pipeline exists. More recently, inhaled probiotics, although technically challenging from a formulation perspective, have shown promising results in preclinical models, including inhibition of lung cancer metastasis (Le Noci et al., 2018) and eradication of *Pa*, either alone (Glieca et al., 2024; Tran et al., 2024) or in combination with phages (Byun et al., 2026). Beyond the nature of these interactions, the timing may reveal also critical, with early-life modulation of the microbiome, such as by using probiotics in infant formula, potentially having a modest preventive effect on atopic dermatitis by two years of age (Wang et al., 2025). Crosstalk between microbial communities and mucosal immunity, in particular the IgA–pIgR axis, is also essential for maintaining epithelium barrier homeostasis. Disruption of this axis, together with microbiome dysbiosis, is observed in several chronic respiratory diseases (**Figure 3**, **Table 1**) and further compromise mucosal defense. These alterations highlight potential therapeutic opportunities targeting IgA biology, as recently reviewed (Sánchez Montalvo et al., 2022; Bohländer, 2023). Such approaches include passive administration of exogenous (S-)IgA; enhancement of effective endogenous (S-)IgA production or epithelial transcytosis; activation of IgA-producing B cells; modulation of (specific) circulating IgA levels and immunomodulatory strategies, notably involving S-IgA immune complexes. As for microbiome modulation, the timing of such interventions is likely crucial. Experimental rodent studies suggested that S-IgA delivered *in utero* (into the amniotic fluid) can reach the fetal intestine as well as the lungs, bind bacteria after birth, and potentially protect preterm neonates from necrotizing enterocolitis (Whitlock et al., 2023; Whitlock et al., 2024), highlighting the potential of prenatal or perinatal IgA-based interventions for both intestinal and lung mucosal immunity. Another key player within this system is pIgR, which could represent a promising therapeutic delivery pathway. Proof-of-concept studies demonstrated that pIgR can be exploited as a highly selective, unidirectional transcytotic pathway for the delivery of α1-antitrypsin or SARS-CoV-2–neutralizing biologics into the airway lumen (Eckman et al., 1999; Ferkol et al., 2000; White et al., 2021). These approaches rely on bifunctional recombinant proteins in which a pIgR-targeting antibody domain mediates epithelial binding and transcytosis, with preserved biophysical stability and minimal competition with endogenous IgA (Maruthachalam et al., 2020), while a fused biologic part exerts its effect at the airway mucosal surface. Such strategies could theoretically be extended to other protein deficiencies, such as surfactant proteins or to deliver antimicrobials or antibodies directly into the mucosal lumen. These early findings suggest that targeting the pIgR system may open new translational avenues for precision therapies in respiratory diseases.

Another important level of host-microbe interactions is through intersystem crosstalk, as discussed earlier in this review. Immune and microbial signals can circulate between distant compartments, linking the respiratory and gastrointestinal tracts with neuroimmune pathways and extending to other mucosal sites such as the mammary glands. IgA-producing B cells may migrate between these sites, explaining the presence of gut-derived IgA in the lung or breast. For instance, previously infected and vaccinated mothers show higher anti-spike IgA levels in breast milk than naïve ones, providing additional protection against COVID-19 to infants through breastfeeding (Perretta et al., 2025). However, targeting the appropriate mucosal compartment remains crucial to achieve effective protection. In vaccination studies, the route of immunization determines the level and location of mucosal responses, with nasal and inhaled vaccines overcoming intradermal or oral routes to induce respiratory IgA responses and to limit pathogen colonization and viral replication, achieving “sterilizing immunity” (Jeyanathan et al., 2022; Kumar et al., 2025; Lisicka et al., 2025). Among them, the inhaled route may provide the most effective and durable protection, characterized by a predominant IgA_2_ response (Chen et al., 2025).

Despite major advances, several key questions remain. The respiratory microbiome extends beyond bacteria to include archaea, fungi, eukaryotic viruses, and bacteriophages, whose interactions with the host remain largely uncharacterized. Deciphering these interkingdom relationships could uncover new therapeutic targets and guide the development of microbiome-based interventions. The specificity of lung IgA responses remains also unclear. Identifying their antigen targets is crucial as IgA could be either protective or pathogenic, the former being probably predominant while the latter could occur upon multivalent aggregation of myeloid Fcα-receptors (Pasquier et al., 2005). Thus, dysregulated IgA responses have been implicated in autoimmune mechanisms observed in COVID-19 (Sinnberg et al., 2023), IPF (Solomon et al., 2020; Boustani et al., 2022) or CF (Theprungsirikul et al., 2020; Yadav et al., 2020) warranting deeper investigation into their functional roles and relevance for disease pathogenesis.

In conclusion, integrative studies that simultaneously capture most microbial actors and mucosal immune responses, including IgA, are essential. Despite rapid technological progress, comprehensive ecological models that account for multiscale microbiome–immune interactions, tissue-specific niches, and longitudinal dynamics are still largely lacking. Building such frameworks will be key to guiding the development of effective, targeted, and durable microbiome-based therapeutic strategies.
